# Development of Global Consensus Sequence and Analysis of Highly Conserved Domains of the HCV NS5B Prote in

**DOI:** 10.5812/hepatmon.6142

**Published:** 2012-09-25

**Authors:** Yasir Waheed, Umar Saeed, Sadia Anjum, Mohammad Sohail Afzal, Muhammad Ashraf

**Affiliations:** 1 Atta-ur-Rehman School of Applied BioSciences (ASAB), National University of Sciences and Technology (NUST), Islamabad, Pakistan

## Abstract

**Background:**

Hepatitis C virus (HCV) is a plus stranded RNA virus which encodes 10 different genes. The HCV NS5B gene encodes a polymerase, which is responsible for the replication of the virus and is a potential target for the development of antiviral agents. HCV has a high mutation rate and is classified into six major genotypes.

**Objectives:**

The aim of this study was to draw a representing consensus sequence of each HCV genotype, align all six consensus sequences to draw a global consensus sequence and also study the highly conserved residues.

**Materials and Methods:**

236 HCV NS5B sequences, belonging to all six genotypes, reported from all over the world were aligned then a representing phylogenetic tree wasdrawn.

**Results:**

The active site residues D220, D225, D318 and D319, which bind the divalent cations, are highly conserved among all the HCV genotypes. The other catalytic pocket residues, R158, S367, R386, and T390 and R394, which interact with the triphosphate of NTPs, are also highly conserved while T390 is mutated to valine in the genotype 5. The motif B residues G283, T286, T287 and N291, which take part in sugar selection by RdRp, are also highly conserved except for T286 which is mutated to proline in the genotypes 3 and 6. The residues E18, Y191, C274, Y276 and H502, which take part in primer/template interaction, are also high conserved except for H502 which is mutated to serine in genotype 2. High variation in all the six consensus sequences was observed in a 12 amino acid beta hairpin loop, which interacts with the double stranded RNA. Nine different peptides from the highly conserved regions of HCV NS5B protein were drawn which can be used as a peptide vaccine. The HCV NS5B phylogenetic tree shows the clusters of different genotypes and their evolutionary association.

**Conclusions:**

In spite of a high mutation rate in HCV, the residues which are present in the catalytic pocket, sugar selection and template/primer interaction are highly conserved. These are target sites for the development of antiviral agents or peptide vaccines. The phylogenetic analysis suggests that different HCV genotypes have been evolved from the genotype 1a.

## 1. Background

Hepatitis C virus (HCV) was discovered in 1989 as a causative agent of non-A non-B hepatitis which belongs to the Flaviviridae family. About 200 million people are living with HCV, involves about 3.3% of the world’s population (1). Most patients with persistent infection of HCV develop chronic hepatitis, fibrosis and even liver cancer (2, 3). HCV has been classified into different genotypes based on at least 67% similarity of nucleotide sequences. There is a strong association between HCV genotypes and both responses to interferon treatment and the degree of clinical progression of chronic HCV infection (4). HCV has six major genotypes and their distribution patterns depend on geographic area and transmission routes (5). HCV comprises a genome of about 9.6 kb, with a single open reading frame of about 3000 amino acids, flanked by 5’ and 3’ untranslated regions. The HCV 5’NTR is 341 bp long and acts as an internal ribosomal entry site. The HCV polyprotein is cleaved co and posttranslational into 10 different proteins. The structural proteins result from cleavage in the N terminal portion of the polyprotein. Two viral proteases mediate downstream cleavage to produce nonstructural proteins. NS3 acts as a protease and NS5B is an HCV RNA dependent RNA polymerase (6). The HCV NS5B polymerase contains the classic fingers, palm and thumb subdomains of a polymerase. The fingers subdomain interacts with the incoming nucleoside triphosphate, as well as with the template base to which it is paired. The palm subdomain is the catalytic center for the nucleotidyl transfer reaction and the thumb subdomain plays a role in positioning the RNA for initiation and elongation (7). NS5B is a potent target for designing antiviral strategies. Currently no vaccine is available for HCV. Different strategies and concepts for vaccination have been used in the last decade. Many studies have been performed on rodents, chimpanzees and human beings. The first approach used in humankind for HCV vaccination was a peptide-based vaccine. HCV vaccination is based on two different concepts in clinical settings. One concept is the use of a preventive vaccine for healthy people to prevent them from being infected, and the second concept is the use of a therapeutic vaccine for the treatment of already infected patients. Preventive vaccinations against HCV were used to induce an immune response in healthy people, including the generation of antigen-specific T cells (8).

## 2. Objectives

The aim of the present study was to draw a global consensus sequence of the NS5B protein of HCV, study the highly conserved residues and draw a phylogenetic tree.

## 3. Materials and Methods

### 3.1. Drawing Consensus Sequence of HCV NS5B

236 Hepatitis C virus gene sequences were randomly selected from the NCBI belonging to six different genotypes. The sequences were fed into CLC workbench software. The HCV NS5B sequences were trimmed using the NS5B Nzl isolate sequence as the reference. The NS5B sequences were then translated in CLC workbench software and deduced amino acid sequences were obtained. We aligned the HCV NS5B amino acid sequences of each genotype to draw representing consensus sequences of all six genotypes. Sixty genotype 1 sequences belonged to the subtypes 1a, 1b and 1g with accession numbers of AF011751, AM910652, EF407411-EF407413, EF407429, EF407432-EF407464, EF407485-EF407504, reported from the USA, Germany and Spain were used to construct the genotype 1 consensus sequence using CLC workbench software. Fifty two genotype 2 sequences belonged to the subtypes 2a, 2b, 2c, 2i, 2k with accession numbers of HQ639938, HQ639939, HQ639943-HQ639945, JN180460, AB030907, AB559564, AF238486, AY232730-AY232739, AY232742-AY232749, D10988, AB031663, D50409, FJ872250-FJ872253, FJ872259-FJ872261, AB031663, FJ872254-FJ872257 reported from China, Denmark, Indonesia, France and Japan were used to construct the genotype 2 consensus sequence using CLC workbench software. Sixteen genotype 3 sequences belonged to the subtypes 3a, 3b, 3k with accession numbers of AM423012, AM423014, AM423016, D17763, D28917, DQ437509, GQ275355, GU294484, GU814263, HQ639941, HQ639942, HQ738645, HQ912953, AY515261, D49374, D63821 reported from Pakistan, Japan, France, India, Switzerland and Denmark were used to construct the genotype 3 consensus sequence using CLC workbench software. Forty one genotype 4 sequences belonged to the subtypes 4a, 4b, 4c, 4d, 4f, 4g, 4k, 4l, 4m, 4n, 4o, 4p, 4q, 4r, 4t with accession numbers of AY973862-AY973864, DQ418783, DQ418788, DQ418789, DQ516084, FJ872297, FJ872299-FJ872302, GU814265, HM566120, GU814265, HM566120, NC009825, Y11604, FJ462435,FJ462436, DQ418786, DQ516083, EU392172, FJ462437, EF589160, EF589161, EU392169, EU392170, EU392174, EU392175, FJ462432, EU392171, EU392173, FJ462438, FJ872307, FJ839870, FJ462433, FJ462441, FJ462440, FJ462431, FJ462434, FJ462439, FJ839869 reported from Egypt, Canada, the USA, France, Spain, Cameroon and Ireland were used to construct the genotype 4 consensus sequence using CLC workbench software. Seventeen genotype 5 sequences belonged to the subtype 5a with accession numbers of Y13184, FJ272356-FJ272370, AF064490 reported from the USA, France and South Africa were used to construct the genotype 5 consensus sequence using CLC workbench software. Fifty genotype 6 sequences belonged to the subtypes 6a, 6c, 6e, 6g, 6i, 6j, 6k, 6l, 6m, 6p, 6r, 6t, 6u, 6v with accession numbers of Y12083, HQ912955, HQ912954, HQ639936, DQ480512-DQ480524, AY973866, AY973865, AY859526, EF424629, EU408326, EU246932,DQ314806, D63822, EU246935, DQ835770, DQ835762, DQ835769, DQ835761, DQ278893, DQ278891, AY878651, AY878650, EU246933, EF424628, DQ835765-DQ835767, DQ835763, EF424626, DQ314805, EU408328, EF632070, EU408332, EU408330, FJ435090, EU798761, EU798760, EU158186 reported from the USA, China, Hong Kong, Thailand, Vietnam, Japan, the Netherlands, Canada and the UK were used to construct the genotype 6 consensus sequence using CLC workbench software.

### 3.2. Peptides Designing and Phylogenetic Analysis

The consensus sequences of all the six HCV genotypes were drawn in CLC workbench software. These consensus sequences were aligned in the CLC workbench to get the global consensus sequence. The consensus sequence was used to study variations in different motifs and domains of the HCV NS5B. Short peptides from the highly conserved regions of the HCV NS5B protein were selected from the consensus sequence analysis; these peptides are the best targets to be tested as a potential peptide vaccine. To draw a phylogenetic tree of the HCV NS5B gene belonging to different genotypes we used 236 sequences; 60 sequences were from the genotype 1, 52 sequences from the genotype 2, 16 sequences from the genotype 3, 41 sequences from the genotype 4, 17 sequences from the genotype 5 and 50 sequences from the genotype 6. All 236 sequences were first aligned in the CLC workbench software and the aligned file was then subjected to the UPGMA method to draw a phylogenetic tree by UPGMA method.

## 4. Results

**Figure 1:**
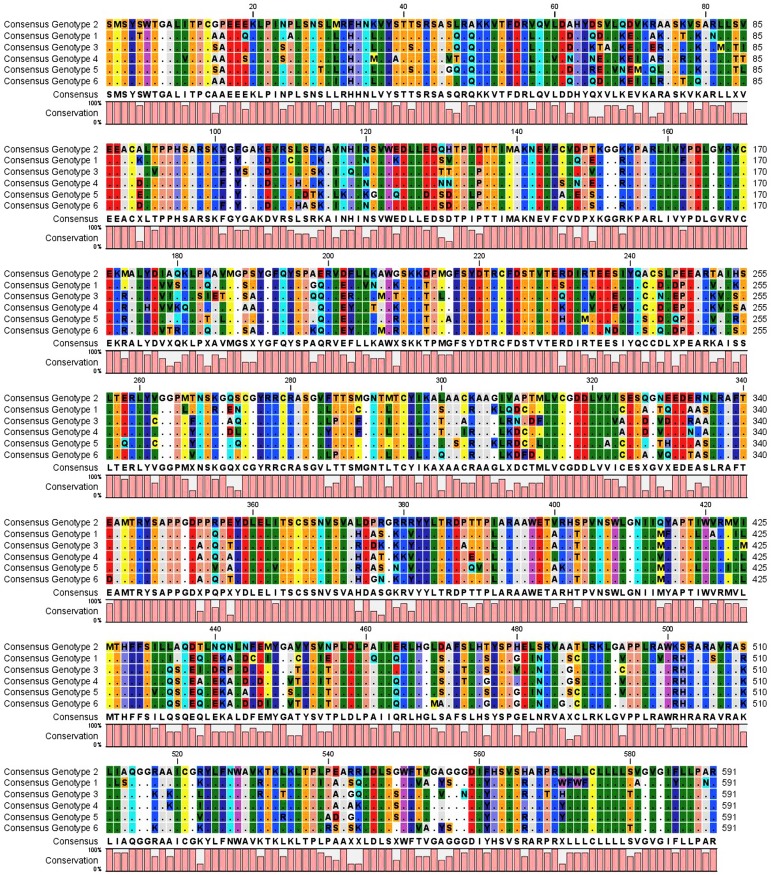
Multiple Sequence Alignment of Consensus Sequences of the Genotypes 1 to 6 of the HCV Ns5b Protein Are Shown

**Figure 2:**
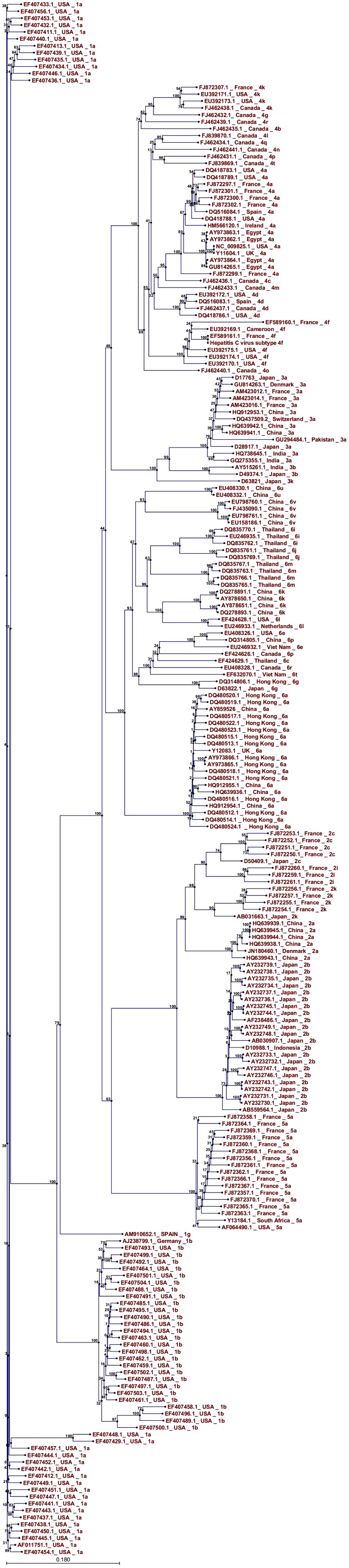
Phylogenetic Tree of 236 HCV NS5B Sequences Belonging to the all Six Genotypes Was Constructed in CLC Workbench Software by the UPGMA Method

We have drawn the HCV NS5B consensus sequence of each HCV genotype. All the consensus sequences were aligned to study the residues which were highly conserved among all the genotypes. Figure 1 shows the alignment of the consensus sequence of all the six HCV genotypes; the global consensus sequence is shown at the base. Conserved residues are shown with their corresponding symbols while the highly variable amino acids are denoted by “x” symbol. The alignment of all the consensus sequences will help us to study the highly conserved residues in the HCV NS5B protein. Short peptides of 9 to 18 amino acids were designed from the highly conserved regions of the HCV NS5B consensus protein sequences; the sequence and position of these peptides are shown in Table 1. These are the positions which are highly conserved and are the targets to design peptide vaccines or site specific inhibitors. A phylogenetic tree of 236 HCV NS5B sequences belonging to the all six genotypes reported from all over the world was constructed using the UPGMA method in CLC work bench software as shown in Figure 2. A default value of 100 was used in bootstrap analysis and the values are present at each branch. Sequences from different genotypes are clustered together. The tree shows that the different HCV genotypes have been evolved from the genotype 1a.

**Table 1:** Position and Sequence of the Peptides Which Can be Used as a Peptide Vaccine

**Position of Peptides**	**Sequence of Peptides**
**1-14 **	SMSYSWTGALITPC
**91-99 **	LTPPHSARS
**136-145 **	TTIMAKNEVF
**155-172 **	KPARLIVYPDLGVRVCEK
**217-230 **	FSYDTRCFDSTVTE
**274-284 **	CGYRRCRASGV
**314-322 **	LVCGDDLVV
**336-352 **	LRAFTEAMTRYSAPPGD
**358-373 **	DLELITSCSSNVSVA

## 5. Discussion

The HCV NS5B protein contains palm, fingers and thumb subdomains. The palm region contains five different motifs A to E, which play a major role in the polymerization ability of HCV polymerase. Motif A contains 212 to 234 amino acids, including the D220-X4-D225 region, which forms the catalytic pocket. D220 and D225 are the residues which are responsible for binding with the magnesium ions. Mutations of D220 to glycine or cysteine completely abolish the NS5B function (9-11). Consensus sequence analysis shows that this region is highly conserved among all the HCV genotypes. Motif B contains G283, T286, T287 and N291 and takes part in sugar selection by RdRp (10). The consensus sequence alignment shows that G283, T287 and N291 are highly conserved among all the genotypes while T286 is mutated to proline in genotype 3 and 6. It is reported that the mutation in G283 and T287 completely abolish the HCV NS5B function (9, 10). Motif C contains the highly conserved GDD motif; the consensus sequence alignment shows that this motif is highly conserved among all the genotypes of HCV. The first aspartate binds the second divalent cation and mutation in this motif is not tolerated, resulting in the abolishment of RdRp function (10). Motif D contains 326 to 347 amino acids which forms the palm core structure. Consensus sequence analysis shows that R345 is highly conserved among all the HCV genotypes; mutation of arginine to lysine increases the RdRp activity to 152% compared to the wild type NS5B (9, 10). Motif E contains 360 to 376 hydrophobic amino acids which forms the interaction of palm with thumb. Consensus sequence analysis shows that this motif is highly conserved among all the HCV genotypes. Consensus sequence analysis shows that the catalytic pocket residues D220, D225, D318, D319, which are responsible for binding with divalent cations, are highly conserved. The other catalytic pocket residues R158, S367, R386, T390 and R394, which interact with NTP triphosphates (11), are highly conserved among all the HCV NS5B consensus sequences except for the T390 which is mutated to valine in the genotype 5. A 12 amino acid long beta hairpin loop is present in the HCV NS5B protein which protrudes from the enzyme active site. This loop interferes with binding to the double stranded RNA due to steric hindrance (11, 12). The consensus sequence analysis shows that this loop is highly variable among all the HCV genotypes. It is reported that E18, Y191, C274, Y276 and H502 are important for interaction of template/primer (11). Consensus sequence analysis shows that these residues are highly conserved among all the genotypes of HCV except for the H502 which is mutated to serine in the genotype 2. Also D225, R48, R158, R386, R394 and S367 are the amino acids which interact with the initiating GTP (13); consensus sequence analysis shows that these residues are highly conserved among all six HCV genotypes. In this study we have drawn a phylogenetic tree of 236 HCV NS5B sequences reported from different countries of the world. The tree was constructed by the UPGMA method as shown in Figure 2. The tree show that the genotype 1a occupies the root of the tree, and the first genotype evolved from the genotype 1a was the genotype 1b. The genotype 1b bifurcates in two wings; from one wing the genotypes 3, 4 and 6 evolved and from the second the genotypes 2 and 5. Sarwer et al., drew a phylogenetic tree of 346 HCV NS4a sequences reported from all over the world (14) and reported that different HCV genotypes have been evolved from the genotype 1b, while our study suggests evolution from the genotype 1a. The difference may be due to the less variation in HCV NS4a sequences compared to the HCV NS5B sequences. Our previous study of some core sequence phylogenetic analyses suggested that the Pakistani core sequences have evolutionary associations with sequences reported from Japan (15). The variation in phylogenetic analysis of different HCV genes might be due to different sequence variations and mutation patterns. Our study suggests that there are certain stretches of amino acids which take part in binding with divalent cations, sugar selection and template/primer interaction and are highly conserved. These conserved residues are a potential target for the development of antiviral agents or peptide vaccines. Phylogenetic analysis suggests that different HCV genotypes have been evolved from the genotype 1a.
